# A Prediction Model for ROS1-Rearranged Lung Adenocarcinomas based on Histologic Features

**DOI:** 10.1371/journal.pone.0161861

**Published:** 2016-09-20

**Authors:** Jianya Zhou, Jing Zhao, Jing Zheng, Mei Kong, Ke Sun, Bo Wang, Xi Chen, Wei Ding, Jianying Zhou

**Affiliations:** 1 Department of Respiratory Disease, Thoracic Disease Center, The First Affiliated Hospital, College of Medicine, Zhejiang University, Hangzhou, China; 2 Department of Pathology, The First Affiliated Hospital, College of Medicine, Zhejiang University, Hangzhou, China; Catalan Institute of Oncology, SPAIN

## Abstract

**Aims:**

To identify the clinical and histological characteristics of *ROS1*-rearranged non-small-cell lung carcinomas (NSCLCs) and build a prediction model to prescreen suitable patients for molecular testing.

**Methods and Results:**

We identified 27 cases of *ROS1*-rearranged lung adenocarcinomas in 1165 patients with NSCLCs confirmed by real-time PCR and FISH and performed univariate and multivariate analyses to identify predictive factors associated with *ROS1* rearrangement and finally developed prediction model. Detected with ROS1 immunochemistry, 59 cases of 1165 patients had a certain degree of ROS1 expression. Among these cases, 19 cases (68%, 19/28) with 3+ and 8 cases (47%, 8/17) with 2+ staining were *ROS1* rearrangement verified by real-time PCR and FISH. In the resected group, the acinar-predominant growth pattern was the most commonly observed (57%, 8/14), while in the biopsy group, solid patterns were the most frequently observed (78%, 7/13). Based on multiple logistic regression analysis, we determined that female sex, cribriform structure and the presence of psammoma body were the three most powerful indicators of *ROS1* rearrangement, and we have developed a predictive model for the presence of *ROS1* rearrangements in lung adenocarcinomas.

**Conclusions:**

Female, cribriform structure and presence of psammoma body were the three most powerful indicator of *ROS1* rearrangement status, and predictive formula was helpful in screening *ROS1*-rearranged NSCLC, especially for ROS1 immunochemistry equivocal cases.

## Introduction

Lung carcinomas are the leading cause of cancer-related mortality worldwide and are responsible for 1.4 million fatalities per year [1]. Over the last decade, the development of targeted therapy has prompted efforts to genetically classify patients with lung carcinomas into subsets, such as *epidermal growth factor receptor* (*EGFR*)-mutated type [2][3], and *anaplastic lymphoma receptor tyrosine kinase* (*ALK*)-rearranged type [4]. *ROS1*-rearranged NSCLCs have recently been defined as a new subset of NSCLCs [5]. In phase I clinical trial of crizotinib, strong antitumor activity has been observed in patients with advanced *ROS1*-rearranged NSCLC [6]. These results underline the importance of identifying patients with *ROS1*-rearranged NSCLCs.

Although FISH has been the gold standard method for the detection of *ROS1* rearrangement, its high cost and the high level of expertise and specialized equipment have made it impractical to test every patient with NSCLC. And IHC for ROS1 protein seems to be a promising screening modality, but it was reported that the D4D6 clone may exhibit cross-reactivity with other epitopes such as Her-2, and it also can react in proliferating non-neoplastic pneumocytes [7, 8]. Therefore, identifying the independent predictors for *ROS1*-rearranged NSCLCs and building a prediction model may help pathologists prescreen suitable patients fast and effectively for molecular testing and targeted treatment, especially for IHC equivocal cases. The clinical and histological characteristics of *ROS1* rearrangement have been reported by some studies [9–13], but the significant prediction factors have not been identified because of the paucity of *ROS1*-rearranged samples.

In this study, we analyzed 27 patients with *ROS1* rearrangement in 1165 unselected patients with NSCLCs and compared their clinical features and histological characteristics with those of other subtypes (*EGFR* mutation, *ALK* rearrangement and triple-negative cases). Furthermore, we identified independent predictors for *ROS1* rearrangement, built prediction model for *ROS1*-rearranged NSCLCs by multiple logistic regression analysis, and tested the performance of prediction model on a new cohort of 57 lung adenocarcinomas (53 ROS1- and 4 ROS1+).

## Materials and Methods

### Patients and samples

**Fig 1:**
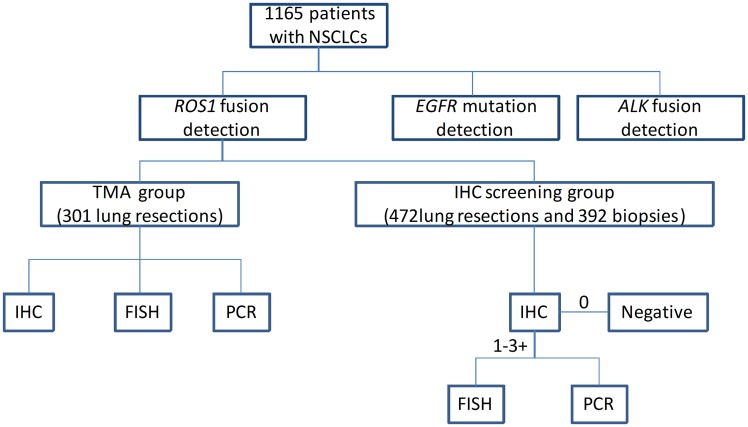
The flow chart of our study.

Our study cohort consisted of 1165 NSCLCs diagnosed between 2010 and 2014. The clinical characteristics of all patients are summarized in S1 Table and the flow chart is present in Fig 1. All the cases were analyzed by real-time PCR for *EGFR* mutation analysis, immunohistochemistry (IHC) (the D5F3 antibody) for *ALK* rearrangement, and positive cases were further evaluated by FISH. Among these 1165 NSCLCs, 301 cases of patients with surgically resected lung adenocarcinoma diagnosed between 2010 and 2012 were made into tissue microarrays (TMA), which were prepared using 2.0-mm cores sampled from 2 different sites of each tumor specimens (Hengtai, Liaoning, China). *ROS1* rearrangement analysis was performed on the TMA by IHC using the D4D6 antibody and then all 301 cases were verified by FISH and real-time PCR; other 864 cases including 472 lung resections and 392 biopsy specimens were detected using the D4D6 antibody, and samples with 1–3+ were verified by FISH and real-time PCR. In order to clarify clinical characteristics of different subtypes, samples with available medical records and genotype were separated into four groups: *EGFR*-mutated, *ALK*-rearranged, *ROS1*-rearranged and triple-negative groups. The validated cohort consisted of 53 ROS1- and 4 ROS1+.Of these, 53 ROS1- were IHC 1–3+ cases screened from 949 surgical NSCLCs diagnosed between 2014 and 2016.

The study was approved by the Ethics Committee of the First Affiliated Hospital of Zhejiang University. The Ethics Committee waived the need for consent for the use of the samples in this research study. None of the cases were selected, but some cases were patients with known genotypes referred by other local hospitals, some of whom had a poor response following a variety of treatments.

### Pathology Review

Haematoxylin and eosin (H&E)-stained slides of all samples were reviewed by two experienced pathologists to evaluate the histologic characteristics based on the IASLC/ATS/ERS classification of lung adenocarcinoma [14].

### Immunohistochemistry

**Fig 2:**
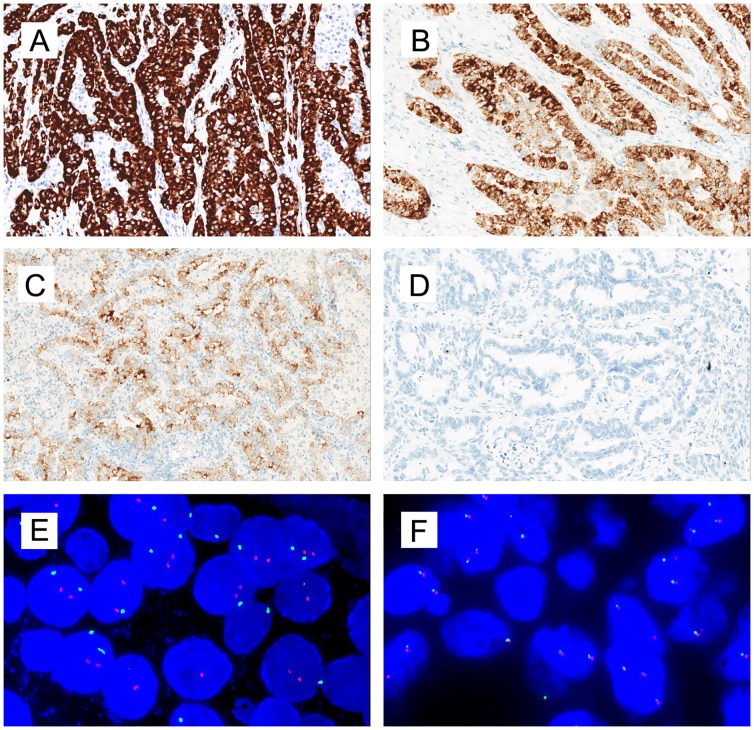
Representative images of ROS1 IHC and FISH results. A, IHC score 3+ for strong, granular cytoplasmic staining in most tumor cells with a diffusely homogenous distribution. B, IHC score 2+ for moderate, smooth cytoplasmic staining with occasional strong staining. C, IHC score 1+ for faint, focal cytoplasmic staining less than the 2+ criteria; D, IHC score 0 for complete absence of staining; E, ROS1 FISH result using break-apart probes. The split green 5’ and orange 3’ signals indicated the presence of *ROS1* rearrangement. F, ROS1 wild type.

IHC for ROS1 was performed on 4 μm-thick FFPE tissues, using rabbit primary monoclonal ROS1 antibody D4D6 (Cell Signaling Technology, Danvers, MA, USA) with a Ventana automated immunostainer (Ventana Medical Systems, Tucson, AZ) according to the manufacturer’s protocol (S1 file). IHC staining scores for ROS1 were assessed as follows: score 3+ for strong, granular cytoplasmic staining diffusely and homogenously in most tumor cells (Fig 2A); score 2+ for moderate, smooth cytoplasmic staining in most tumor cells with occasional strong staining (Fig 2B); score 1+ for faint, focal cytoplasmic staining less than the score 2+ criteria (Fig 2C); and score 0 for the complete absence of staining (Fig 2D). IHC scoring was performed by three pathologists unaware of the FISH results.

### Fluorescence *In Situ* Hybridization

Slices of FFPE tissues, 4μm thick, were used to evaluate the presence of *ROS1* gene fusion by FISH, using a break-apart probe for *ROS1* (6q22 ROS1Break Apart FISH Probe; Abbott Molecular, Des Plaines, IL, USA) according to the technical instructions and interpretation standard (Fig 2E and 2F).

### Real-time PCR

*ROS1* was amplified by multiplex real-time PCRs using a Stratagene Mx3000P real-time PCR system (Stratagene, CA) with an AmoyDx^®^ *ROS1* fusion gene detection kit (Amoy Diagnostics Co., Ltd, Xiamen, China) (S2 file). The status of the *EGFR* mutation and *ALK* rearrangement were also analyzed by real-time PCR and IHC, according to methods previously described [15].

### Statistical Analysis

Statistical analysis was performed using SPSS software version 17.0 (SPSS Inc., Chicago, IL). Continuous variables (age, smoking status and stage) were analyzed using variance analysis. The categorized variables (sex, CEA and histological features) were analyzed using two-tailed the Pearson χ^2^ test, and *P*<0.05 was considered statistically significant. Statistically significant factors for *ROS1* rearrangement derived from univariate analysis (the following variables with *P* values< 0.1: age, sex, smoking status, stage, any solid pattern, any papillary pattern, cribriform structure, extracellular mucus, signet-ring cells, psammoma body) were selected for multivariate analysis using multiple logistic regression and get the predictive model. The efficiency of the prediction model was evaluated by use of the area under the ROC curve.

## Results

### IHC and FISH

59 cases out of 1165 cases tested had a certain degree of ROS1 expression, including 28 with 3+, 17 with 2+, and 14 with 1+ staining. Only 19 cases (68%, 19/28) with 3+ and 8 cases (47%, 8/17) with 2+ staining showed *ROS1* rearrangement by real-time PCR and FISH; the other 32 cases with IHC 1+ to 3+ staining were ROS1 wild type. 290 cases with 0 staining were all wild type verified by FISH and real-time PCR. If both 2+ and 3+ ROS1 protein expression is considered positive, ROS1 IHC is 100% sensitive and 94.4% specific for the presence of *ROS1* rearrangement by FISH (S2 Table).

### Clinical Characteristics of patients with *ROS1*-rearranged NSCLCs

The clinical features of patients with *ROS1*-rearranged, *ALK*-rearranged, *EGFR*-mutated, and triple-negative tumors are summarized in Table 1. The characteristics of patients with *ROS1* rearrangement were female, younger in age, never or light smokers and in a more advanced stage at diagnosis, which was similar with *ALK*-rearranged patients. Compared with the *ROS1*-rearranged patients (average, 53 years), *EGFR*-mutated (average, 60 years) and triple-negative patients (average, 60 years) were older (*P* = 0.004). The *EGFR*-mutated patients tended to occur in patients of lower stage at diagnosis (*P* = 0.049) compared with patients with *ROS1* rearrangement. The triple-negative NSCLCs were found significantly more commonly in males (63.8%, *P*<0.001) and smokers (48%, *P* = 0.008).

**Table 1:** Comparison of clinicopathologic parameters among *ROS1* rearrangement, *ALK* rearrangement, *EGFR* mutation and triple-negative lung carcinomas.

		ROS1+	ALK +	EGFR+	Triple-negative	*P-value*		
						ROS+ vs.ALK+	*ROS+ vs*.*EGFR+*	*ROS+ vs*.*Triple-negative*
NO.		27	67	377	301			
Clinical findings								
Age(Average)		53(27–78)	52(23–77)	60(27–87)	60(26–83)	0.776	0.004*	0.004*
Sex(M:F)		8:19	32:35	144:233	192: 109	0.166	0.418	<0.001*
Smoking	0	82%	70%	74%	51%	0.481	0.739	0.008*
	<20	7%	16%	9%	10%			
	≥20	11%	12%	16%	38%			
	Unknown	0%	1%	1%	1%			
CEA	≤5	70%	54%	50%	52%	0.473	0.159	0.221
	>5	30%	34%	40%	40%			
	Unknown	0	12%	10%	8%			
Stage(I:II:III:IV)		4:3:8:12	6:13:25:21	81:95:103:82	61:65:74:90	0.455	0.049*	0.324
	Unknown	0	2	16	11			
Histomorphology of resected samples								
NO.		14	38	82	62			
Lepidic predominant		7%	3%	16%	19%	0.470	0.685	0.441
Acinar predominant		57%	47%	59%	34%	0.755	1.000	0.133
Papillary predominant		14%	13%	12%	8%	1.000	0.686	0.606
Solid predominant		21%	37%	13%	37%	0.341	0.424	0.357
Any solid pattern		93%	74%	32%	50%	0.251	<0.001*	0.003*
Any papillary pattern		43%	29%	22%	13%	0.506	0.107	0.018*
Any lepidic pattern		29%	11%	38%	32%	0.189	0.565	1.000
Any acinar pattern		86%	68%	76%	86%	0.300	0.511	1.000
Cribriform feature		86%	58%	22%	15%	0.100	<0.001*	<0.001*
Extracellular mucus		71%	68%	20%	18%	1.000	<0.001*	<0.001*
Signet-ring cells		21%	29%	1%	5%	0.732	0.009*	0.072
Psammoma body		43%	11%	4%	5%	0.016*	<0.001	<0.001*
perinuclear vacuole		57%	66%	50%	29%	0.746	0.774	0.063
Hepatoid cell		7%	21%	5%	3%	0.415	0.554	0.462

F indicates female; M, male.

* marks parameters showing statistical significance by univariate analysis.

** Only resected samples were evaluated for the histologic characteristics.

### Histologic Characteristics of *ROS1*-rearranged NSCLCs

**Fig 3:**
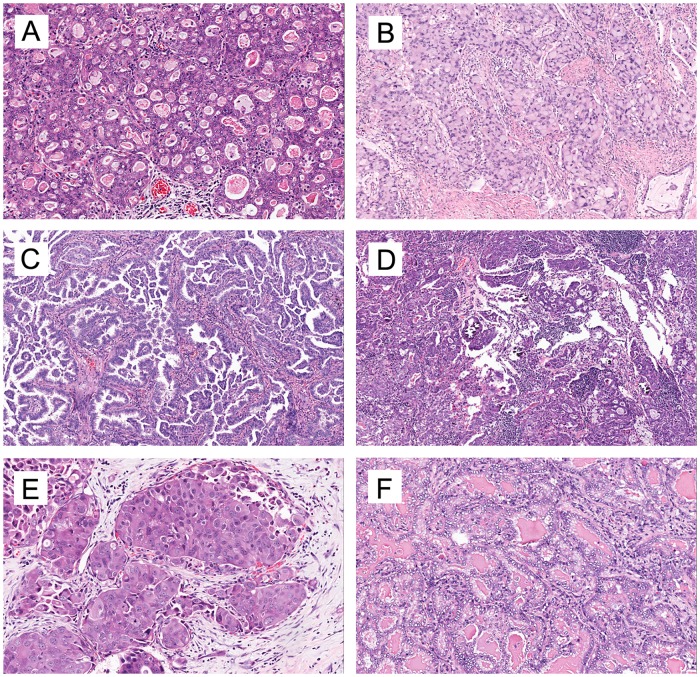
Representative growth patterns and cellular features of *ROS1*-rearranged lung adenocarcinoma. Cribriform structure (A), solid pattern with signet-ring cells (B), and papillary growth pattern (C). Psammomatous calcifications are common in *ROS1*-rearranged lung adenocarcinoma (D). Moreover, *ROS1*-rearranged lung adenocarcinoma has distinct cytologic features: hepatoid tumor cells (E) and perinuclear vacuole (F). A-D and F were taken under 100× magnification and E under 200 × magnifications. Tissues from metastases were available for 9 of the *ROS1*-rearranged cases. The histologic and cytologic features of the metastatic tumors were similar to those of the primary site: the same growth pattern, nuclear features and psammoma body were present in both primary and metastatic tumors (S1 Fig). In addition, tumors at metastatic sites were ROS1 positive with D4D6 staining as observed at the primary sites.

Most *ROS1*-rearranged tumors were adenocarcinoma, and one case had focal squamous differentiation (accounting for 10%of the tumor volume). The histologic characteristics of 27 *ROS1*-rearranged NSCLCs are described in detail in S3 Table. These cases consisted of 14 resected specimens and 13 biopsy specimens. In the resected group, the acinar-predominant growth pattern was the most commonly observed in 57% (8/14) of *ROS1*-rearranged tumors, followed by the solid pattern (21%) and papillary pattern (14%) (Fig 3A–3C). All 8 acinar-predominant cases showed a cribriform structure, 7 of which were found to have abundant extracellular mucus or signet-ring cells. Cribriform structure, extracellular mucus, and psammomatous calcifications (Fig 3D) were frequently noted in 85.7% (12/14), 71.4% (10/14) and 42.9% (6/14) of *ROS1*-rearranged tumors, respectively. In the biopsy group, solid patterns were the most frequently observed with 7 cases (78%), and 3 of them (33%) presented signet-ring cells. Moreover, *ROS1*-rearranged tumors were found to be associated with distinct cytologic features: 1. 56% (5/9) of biopsy specimens were composed of “hepatoid” cells (Fig 3E), which has eosinophilic cytoplasm, round nuclei and obvious nucleoli; 2. Tumor cells in 57% (8/14) of resected specimens showed distinct nuclei with vacuole around nuclei and prominent nucleoli (Fig 3F).

### Comparison between *ROS1*-positive and *ROS1*-negative cases

Statistical analysis was performed in resected samples, and histologic features of 14 *ROS1*-rearranged NSCLCs were compared with those of 182 *ROS1*-negative NSCLCs (Table 2). Cribriform feature (*P*<0.001), psammoma body (*P*<0.001), any solid pattern (*P* = 0.001) and mucinous cells or extracellular mucus (*P* = 0.003) were associated with *ROS1*- rearranged NSCLCs.

**Table 2:** Comparison of histological parameters among *ROS1*-positive and *ROS1*-negative NSCLCs.

	*ROS1*+(n = 14)	*ROS1*-(n = 182)	*P-value*
Lepidic predominant	1	156	0.73
Acinar predominant	8	95	0.5
Papillary predominant	2	162	1
Solid predominant	3	134	0.928
Any solid pattern	13	97	0.001*
Any papillary pattern	6	145	0.104
Any lepidic pattern	4	127	1
Any acinar pattern	12	41	0.702
Cribriform feature	12	133	0*
Mucinous cells or extracellular mucus	10	129	0.003*
signet-ring cells	3	167	0.244
Psammoma body	6	172	0*
Perinuclear vacuole	8	98	0.427
Hepatoid cell	1	168	1

* marks parameters showing statistical significance by univariate analysis.

### Comparison among *ROS1*-positive, *ALK*-positive, *EGFR*-mutated and triple-negative NSCLCs

The histologic characteristics of the four groups of patients are compared in Table 1. Similar with the clinical features, the histologic characteristics of *ROS1*-rearranged patients were similar as those of *ALK*-rearranged patients but very different from the *EGFR*-mutated and triple-negative NSCLCs. Cribriform structure was seen in 86% of *ROS1*+ tumors (compared with only 15% of triple-negative cases and 22% *EGFR*-mutated NSCLCs, both *P*<0.001) and extracellular mucus was seen in 71% of *ROS1*+ tumors (compared with only 18% of triple-negative cases and 20% *EGFR*-mutated NSCLCs, both *P*<0.001). Signet-ring cells was seen in 21% of *ROS1*+ tumors (compared with only 1% of *EGFR*-mutated NSCLCs, *P* = 0.009). Any papillary pattern (43%) and any solid pattern (93%) were both frequently seen in *ROS1*+ tumors (compared with only 13% of triple-negative NSCLCs and 32% of *EGFR*-mutated NSCLCs, *P* = 0.009 and *P*<0.001). Notably, the psammoma body (Fig 3D) is more likely to be present in *ROS1*-rearranged cases (43%, 6/14) than in *ALK*-rearranged cases (11%, 4/38) (*P* = 0.016), and even fewer in *EGFR*-mutated (4%, 3/82) and triple-negative (5%, 3/62) cases.

### Multiple Logistic Regression Analysis

We performed multiple logistic regression analysis to identify the independent predictors for *ROS1* rearrangement status. No significant differences were found with patient’s age, smoking status, lepidic predominant, acinar predominant, papillary predominant, solid predominant, the presence of perinuclear vacuole, hepatoid cell, extracellular mucus and signet-ring cells. The three criteria (female sex, the presence of psammoma body and cribriform structure) were identified as independent predictors for *ROS1* rearrangement. The significant prediction factors for *ROS1* rearrangement are shown in Table 3. Of these predictors, the presence of a cribriform structure had the highest risk ratio (odds ratio, 14.415; 95% confidence interval, 2.849–72.938; *P* = 0.001). Based upon the result of a multiple logistic regression analysis, we developed a prediction model for *ROS1* rearrangement: logit(P) = -5.743 + 1.845 × [sex] + 2.668 × [cribriform structure] + 2.443 × [psammoma body], where “1” indicates the presence of cribriform structure, psammoma body and male, “0” indicates the absence of cribriform structure, psammoma body and female sex. S2 Fig is the ROC curve for the prediction model; the area under the curve is 0.889, indicating that this prediction model was clinically valuable for the prediction of *ROS1* rearrangement. In order to be suitable for clinical routine use, prediction model were simplified according to the result of multiple logistic regression analysis: female = 1, male = 0, cribriform structure = 2, and psammoma body = 2, total scores of 2 or more are considered “highly probable” for *ROS1* rearrangement. We tested the prediction model on additional cohort of 57 lung adenocarcinoma including 4 ROS1+ and 53 ROS1 IHC 1–3+ but FISH negative cases. Finally we get 7 “highly probable” cases, and 4 of them were verified ROS1 positive by FISH. The prediction model performed with sensitivity of 100% and specificity of 94.3%.

**Table 3:** The Result of Multiple Logistic Regression Analysis.

Variables	β-Coefficient	SE	Wald Test	*p*-Value	OR	95%CI
Sex (male = 1, female = 0)	1.845	0.769	5.765	0.016	6.331	1.404–28.552
Cribriform feature (yes = 1,no = 0)	2.668	0.827	10.404	0.001	14.415	2.849–72.938
Psammoma body (yes = 1,no = 0)	2.443	0.786	9.664	0.002	11.512	2.467–53.727
Constant	-5.743	1.035	30.817	0.000	0.003	

## Discussion

In this study, we identified 27 *ROS1*-rearranged NSCLCs. *ROS1*-rearranged NSCLCs has distinct pathologic features. Acinar-predominant growth pattern with cribriform structure was the most common histologic characteristics in the resected group, which was in agreement with what Chen et al [8,9] reported. In the advanced stage group, the solid-predominant growth pattern with signet-ring cells was the most common feature, which was consistent with a previous report [12]. This difference between resection samples and biopsy samples may due to the different stages of the patients, as most patients of resected group were in stage I-IIIA whereas all patients with biopsy samples were in stage IV. Additionally, the tumors of advanced stage showed a trend toward presentation with a solid pattern. Due to the paucity of *ROS1*-rearranged NSCLCs, the characteristic morphologic appearances of *ROS1*-rearranged NSCLCs remained has discrepancy. Sholl et al reported that solid and papillary-predominant patterns were more common than the acinar pattern [7]. In contrast, Go et al found that solid and papillary-pattern growth were the two most common growth patterns in *ROS1*-rearranged NSCLCs [12]. We presumed that this discrepancy may be due to the mix of biopsy samples, the small number of cases and the different stages of the patients. Our histologic analysis of the different specimen types may help elucidate the previous controversy.

In our study, female sex, the presence of psammoma body and cribriform structure were identified as independent predictors for *ROS1* rearrangement. The presence of cribriform structure was the most significant independent characteristic of *ROS1* rearrangement. Cribriform architecture was considered as a pattern of acinar adenocarcinoma [14], and it was defined by invasive “back-to-back” glands with poorly formed glandular spaces lacking intervening stroma or having very thin stroma in limited areas between glandular spaces [16,17]. Yoshida et al identified presence of mucinous cribriform architecture or solid growth with signet-ring cells in 53% of the *ROS1*-rearranged cases, which was similar with our cohort [11]. However, the statistical analysis for histologic features was not performed in their cohort. We finally identify cribriform structure but not signet-ring cell (*P* = 0.538) was a predictor for *ROS1*-rearranged NSCLCs by multivariate analysis. The extracellular mucus was also not identified as a predictor for *ROS1*-rearranged NSCLCs by multivariate analysis, although it was significantly correlated with *ROS1* rearrangement (*P* = 0.003) by χ^2^ test. Recently, Kadotaet al found cribriform predominant tumors was a poor prognostic subtype of acinar predominant tumors, and presence (≥10%) of the cribriform component is an independent predictor of recurrence [18]. This was concordance with the poor prognosis of *ROS1*-rearranged patients [9,10]. It would be interesting to find out the proportions of *ROS1* rearrangement in NSCLCs with cribriform component.

The psammoma body was also identified as an independent characteristic of *ROS1* rearrangement. The psammoma body was usually present in papillary thyroid carcinoma [19]. In our cohort, psammoma body were noted in 43% of *ROS1*-rearranged NSCLCs but in few cases (11%) of *ALK*-rearranged NSCLCs (*P* = 0.016) and even fewer cases in the *EGFR*-mutated (4%) and triple negative tumors (5%). Sholl et al also found that *ROS1*-rearranged NSCLCs had frequent (67%, 6/9) psammomatous calcifications [20]. We advise pathologists to record the presence of psammomatous calcifications in NSCLCs, which may be helpful for prescreening *ROS1*-rearranged patients.

Histomorphological features of the primary tumor site, such as psammoma body, growth pattern, nuclear features and abundant mucus, were also preserved in the metastatic tumors. The metastatic sites of the *ROS1*-rearranged cases also expressed the ROS1 protein, which suggested that the histomorphology of metastatic tumors could be evaluated for identifying patients with *ROS1* rearrangement, and screening for *ROS1* could also be conducted in metastatic tumors on the condition of primary tumors were unavailable.

In this study, 290 IHC 0 and 14 IHC1+ staining were negative verified by FISH, and 19 cases (68%, 19/28) with 3+ and 8 cases (47%, 8/17) with 2+ staining showed *ROS1* rearrangement by real-time PCR and FISH; nine out of twenty-eight tumors (32%) with strong expression of ROS1 protein (3+) and nine out of seventeen 2+ staining were found to be negative by FISH and real-time PCR. Although the non-specific staining has been excluded, such as osteoclast-type giant cells and reactive epithelial proliferations [20], these nine discordant 3+ cases in our study were difficult to interpret because of their strong and diffuse cytoplasmic staining for ROS1. Based on earlier reports, 5 cases with strong expression of ROS1 protein but negative by FISH assay were identified [8,11,20]. Mescam-Mancini suggested that the D4D6 clone may exhibit cross-reactivity with other epitopes such as Her-2 [8]. Understanding the histologic characteristics of *ROS1* rearrangement will help pathologists recognize the *ROS1*-rearranged cases and exclude the false positive cases. Therefore, we built a prediction model by multiple logistic regression analysis to prescreen suitable patients for molecular testing. The prediction model was validated by the ROC curve and the area under ROC curve was 0.899, which was close to maximum 1. We further tested the prediction model on validated cohort, and it turned out to be effective: sensitivity of 100% and specificity of 94.3%. Therefore, this prediction model appeared to be effective to help pathologists identify potential *ROS1*-rearranged patients, especially in IHC equivocal cases.

Some limitations of our study must be considered. 1. *KRAS* mutation group was not included in this study because the incidence of *KRAS* is low (<10%) in the Chinese population [21], and the analysis of *KRAS* mutation is not routinely performed in patients with lung carcinomas. 2. Most of our cases were in newly diagnosed patients in whom survival analysis could not be performed due to the short follow-up period. In conclusion, female sex, a cribriform structure and the presence of psammoma body were characteristics of patients with *ROS1*-rearranged NSCLCs. Combined with IHC, the prediction model of *ROS1*-rearranged NSCLCs may help pathologists find “highly probable” patients before molecular tests. However, none of prediction model can work perfectly, the diagnosis of *ROS1*-rearrangement need to be validated by FISH or real-time PCR.

## Supporting Information

S1 Fig Two representative cases of metastatic lung adenocarcinoma with ROS1 rearrangement. A and B (Case 1) depicts a case with an acinar predominant growth pattern. A was the primary tumor. And B was the metastatic site in a lymph node, both showing the same growth pattern. C and D (Case 2) depicta case with a solid growth pattern with the presence of signet-ring cells. Solid growth pattern, signet-ring cell and psammomatous calcifications were present in both the primary tumor and metastatic tumor. Expression of the ROS1 protein was strongly and diffusely positive in the primary tumor and at metastatic sites in both cases. All pictures were taken under 200× magnification.
(TIF)

S2 Fig The ROC curve of the prediction formula for *ROS1* rearrangement. The area under the curve was 0.889, indicating that this prediction model was valuable for the prediction of *ROS1* rearrangement.
(TIF)

S1 File Immunohistochemistry. (DOC)

S2 File Real-time PCR. (DOCX)

S1 Table Clinical Characteristics of Patients Tested in our Study. (DOC)

S2 Table Sensitivity and Specificity of ROS1 IHC for *ROS1*rearrangement by FISH Calculated on the Basis of 2+ Protein ExpressionCutoff. (DOC)

S3 Table Pathologic Features of *ROS1*-rearranged NSCLCs. (DOC)

## References

[1] Jemal A, Bray F, Center MM, Ferlay J, Ward E, Forman D. Global cancer statistics. CA Cancer J Clin.2011; 61: 69–90. 10.3322/caac.20107 21296855

[2] Paez JG, Janne PA, Lee JC, Tracy S, Greulich H, Gabriel S, et al EGFR mutations in lung cancer: correlation with clinical response to gefitinib therapy. Science. 2004; 304: 1497–1500. 15118125 10.1126/science.1099314

[3] Lynch TJ, Bell DW, Sordella R, Gurubhagavatula S, Okimoto RA, Brannigan BW, et al Activating mutations in the epidermal growth factor receptor underlying responsiveness of non-small-cell lung cancer to gefitinib. N Engl J Med. 2004; 350: 2129–2139. 15118073 10.1056/NEJMoa040938

[4] Kwak EL, Bang YJ, Camidge DR, Shaw AT, Solomon B, Maki RG, et al Anaplastic lymphoma kinase inhibition in non-small-cell lung cancer. N Engl J Med. 2010; 363: 1693–1703. 10.1056/NEJMoa1006448 20979469 PMC3014291

[5] Stumpfova M, Janne PA. Zeroing in on ROS1 rearrangements in non-small cell lung cancer. Clin Cancer Res. 2012; 18: 4222–4224. 10.1158/1078-0432.CCR-12-1812 22859716

[6] Shaw AT O S, Bang YJ, Camidge DR, Camidge DR, Solomon BJ, Salgia R, et al Crizotinib in ROS1-rearranged non-small-cell lung cancer. N Engl J Med. 2014; 371: 1963–1971. 10.1056/NEJMoa1406766 25264305 PMC4264527

[7] Sholl LM, Sun H, Butaney M, Zhang C, Lee C, Janne PA, et al ROS1 immunohistochemistry for detection of ROS1-rearranged lung adenocarcinomas. Am J Surg Pathol. 2013; 37: 1441–1449. 10.1097/PAS.0b013e3182960fa7 23887156 PMC3831351

[8] Mescam-Mancini L, Lantuejoul S, Moro-Sibilot D, Rouquette I, Souquet PJ, Audigier-Valette C, et al On the relevance of a testing algorithm for the detection of ROS1-rearranged lung adenocarcinomas. Lung Cancer. 2014; 83: 168–173. 10.1016/j.lungcan.2013.11.019 24380695

[9] Chen YF, Hsieh MS, Wu SG, Chang YL, Shih JY, Liu YN, et al Clinical and the prognostic characteristics of lung adenocarcinoma patients with ROS1 fusion in comparison with other driver mutations in East Asian populations. J Thorac Oncol. 2014; 9: 1171–1179. 10.1097/JTO.0000000000000232 25157770

[10] Cai W, Li X, Su C, Fan L, Zheng L, Fei K, et al ROS1 fusions in Chinese patients with non-small-cell lung cancer. Annals of Oncology. 2013; 24: 1822–1827. 10.1093/annonc/mdt071 23514723

[11] Yoshida A, Kohno T, Tsuta K, Wakai S, Arai Y, Shimada Y, et al ROS1-rearranged lung cancer: a clinicopathologic and molecular study of 15 surgical cases. Am J Surg Pathol. 2013; 37: 554–562. 10.1097/PAS.0b013e3182758fe6 23426121

[12] Go H, Kim DW, Kim D, Keam B, Kim TM, Lee SH, et al Clinicopathologic analysis of ROS1-rearranged non-small-cell lung cancer and proposal of a diagnostic algorithm. J Thorac Oncol. 2013; 8: 1445–1450. 10.1097/JTO.0b013e3182a4dd6e 24128715

[13] Seung EL, Boram L, Mineui H, Ji-Young S, Kyungsoo J, Maruja E L, et al Comprehensive analysis of RET and ROS1 rearrangement in lung adenocarcinoma. Modern Pathology. 2015; 28: 468–479. 10.1038/modpathol.2014.107 25234288

[14] Travis WD, Brambilla E, Noguchi M, Nicholson AG, Geisinger KR, Yatabe Y, et al International association for the study of lung cancer/american thoracic society/european respiratory society international multidisciplinary classification of lung adenocarcinoma. J Thorac Oncol. 2011; 6: 244–285. 10.1097/JTO.0b013e318206a221 21252716 PMC4513953

[15] Zhou JY, Zhao J, Sun K, Wang B, Wang L, Chen X, et al Accurate and Economical Detection of ALK Positive Lung Adenocarcinoma with Semiquantitative Immunohistochemical Screening. Plos One. 2014; 9. 10.1371/journal.pone.0092828 PMC3965450 24667320

[16] JI E. An update of the Gleason grading system. J Urol. 2010; 183: 433–440. 10.1016/j.juro.2009.10.046 20006878

[17] Egashira Y, Yoshida T, Hirata I, Hamamoto N, Akutagawa H, Takeshita A, et al Analysis of pathological risk factors for lymph node metastasis of submucosal invasive colon cancer. Mod Pathol. 2004; 17: 503–511. 15001992 10.1038/modpathol.3800030

[18] Kadota K, Yeh YC, Sima CS, Rusch VW, Moreira AL, Adusumilli PS, et al The cribriform pattern identifies a subset ofacinar predominant tumors with poor prognosis in patients with stage I lung adenocarcinoma: a conceptual proposal to classify cribriform predominant tumors as adistinct histologic subtype. Modern Pathology. 2013; 1–11. 24186133 10.1038/modpathol.2013.188 PMC4374572

[19] Lee YS, Hong SW, Chang HS, Park CS. Scattered psammomatous calcifications around papillary thyroid carcinoma. World J Surg. 2014; 38: 1738–1742. 10.1007/s00268-014-2460-z 24496808

[20] Sholl LM, Sun H, Butaney M, Zhang C, Lee C, Janne PA, et al ROS1 Immunohistochemistry for Detection of ROS1-Rearranged Lung Adenocarcinomas. Am J Surg Pathol. 2013;37:1441–1449. 10.1097/PAS.0b013e3182960fa7 23887156 PMC3831351

[21] Wen YS, Cai L, Zhang XW, Zhu JF, Zhang ZC, Shao JY, et al Concurrent oncogene mutation profile in Chinese patients with stage Ib lung adenocarcinoma. Medicine. 2014; 93: 296. 10.1097/MD.0000000000000296 PMC4602605 25546673

