# Supplementary figures and images for: A Prediction Model for ROS1-Rearranged Lung Adenocarcinomas based on Histologic Features
Source: PLoS One. 2016 Sep 20;11(9):e0161861. doi: 10.1371/journal.pone.0161861 (PMC5029801; doi:10.1371/journal.pone.0161861)

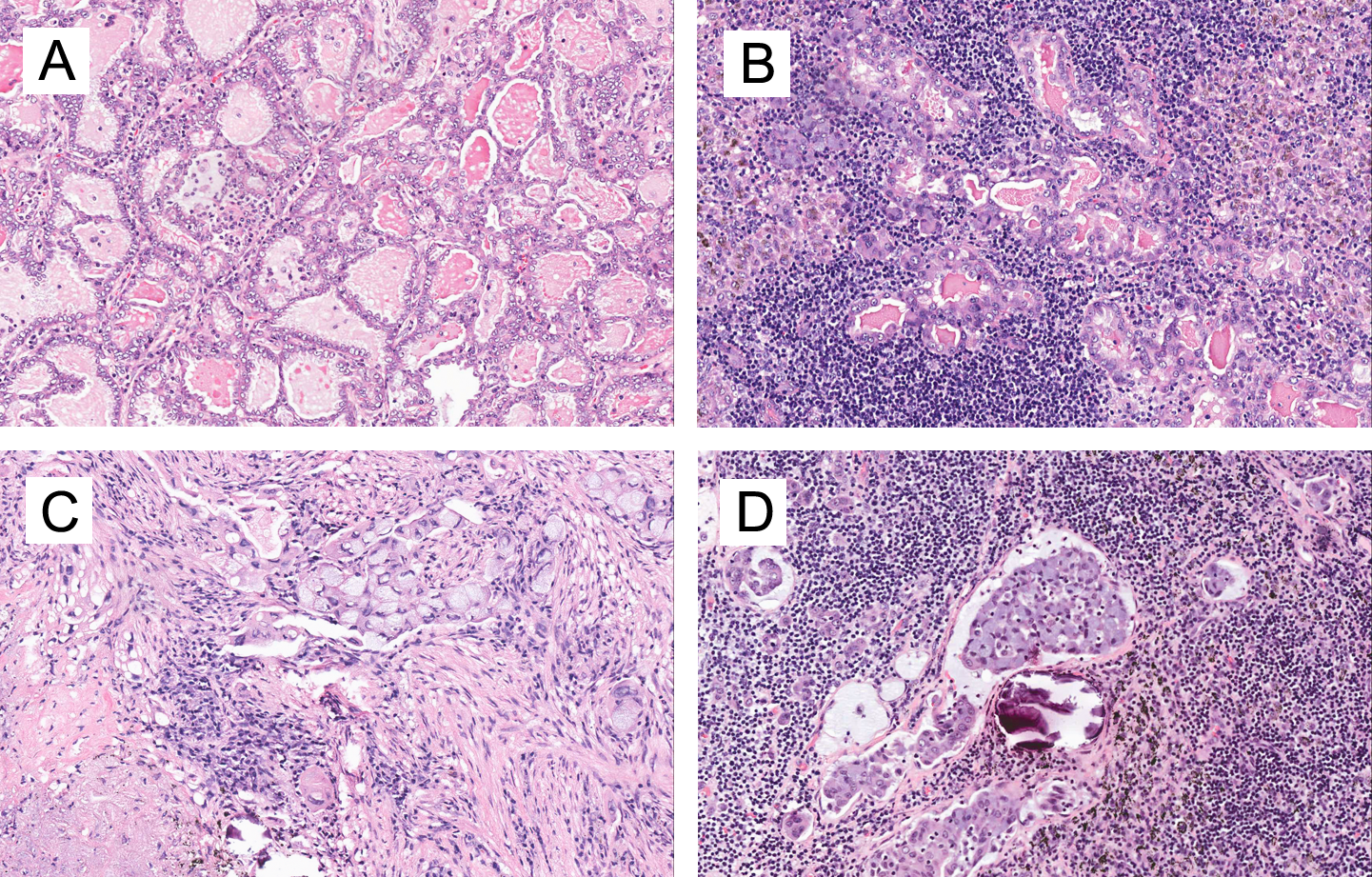

Supplement: S1 Fig — A and B (Case 1) depicts a case with an acinar predominant growth pattern. A was the primary tumor. And B was the metastatic site in a lymph node, both showing the same growth pattern. C and D (Case 2) depicta case with a solid growth pattern with the presence of signet-ring cells. Solid growth pattern, signet-ring cell and psammomatous calcifications were present in both the primary tumor and metastatic tumor. Expression of the ROS1 protein was strongly and diffusely positive in the primary tumor and at metastatic sites in both cases. All pictures were taken under 200× magnification. (TIF) [file pone.0161861.s001.tif]

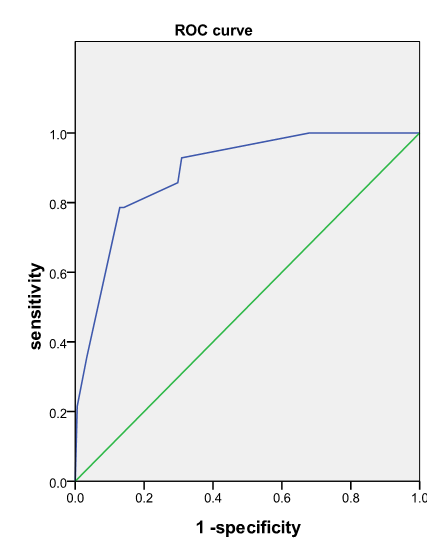

Supplement: S2 Fig — The area under the curve was 0.889, indicating that this prediction model was valuable for the prediction of ROS1 rearrangement. (TIF) [file pone.0161861.s002.tif]
