# Supplementary material for: A Prediction Model for ROS1-Rearranged Lung Adenocarcinomas based on Histologic Features
Source: PLoS One. 2016 Sep 20;11(9):e0161861. doi: 10.1371/journal.pone.0161861 (PMC5029801; doi:10.1371/journal.pone.0161861)
Supplement: S1 File — (DOC) [file pone.0161861.s003.doc]

**Immunohistochemistry**

In brief, the slides were dried at 65°C for 1 hour and deparaffinized using EZ Prep (Ventana Medical Systems) at 75°C for 4 minutes. Antigen retrieval was performed using a CC1 solution for 20 min. Sections were then incubated with the ROS1 D4D6 antibody (1:200) for 24 minutes at 37°C. Signals were detected using optiview amplification kit (Ventana Medical Systems).
