# Supplementary material for: A Prediction Model for ROS1-Rearranged Lung Adenocarcinomas based on Histologic Features
Source: PLoS One. 2016 Sep 20;11(9):e0161861. doi: 10.1371/journal.pone.0161861 (PMC5029801; doi:10.1371/journal.pone.0161861)
Supplement: S2 File — (DOCX) [file pone.0161861.s004.docx]

**Real-time PCR**

The patterns of ROS1 fusions detected in this validation included SLC34A2-ROS1, CD74-ROS1, SDC4-ROS1, EZR-ROS1, TPM3-ROS1, LRIG3-ROS1 and GOPC-ROS1. An internal reference gene (β-actin) and ROS1-rearranged DNA were used as controls. The PCR conditions consisted of one cycle at 95°C for 5 min; 15 cycles of denaturation at 95°C for 25 s, annealing at 64°C for 20 s and elongation at 72°C for 20 s; 31 cycles at 93°C for 25 s, 60°C for 35 s and 72°C for 20 s.
