# Supplementary material for: A Prediction Model for ROS1-Rearranged Lung Adenocarcinomas based on Histologic Features
Source: PLoS One. 2016 Sep 20;11(9):e0161861. doi: 10.1371/journal.pone.0161861 (PMC5029801; doi:10.1371/journal.pone.0161861)
Supplement: S1 Table — (DOC) [file pone.0161861.s005.doc]

Table S1.Clinical Characteristics of Patients Tested in our Study

|  | | | | |
| --- | --- | --- | --- | --- |
|  |  |  |  |  |
|  |  | N=1165 | |  |
| Mean age (range) | | 61 | (23-93) |  |
| Sex | Male | 603 | (52) |  |
|  | Female | 562 | (48) |  |
| Smoking status | |  |  |  |
|  | never | 710 | (61) |  |
|  | Former | 443 | (38) |  |
|  | Unknown | 12 | (1) |  |
| Stage at initial diagnosis | |  |  |  |
|  | I | 279 | (24) |  |
|  | II | 224 | (19) |  |
|  | III | 270 | (23) |  |
|  | IV | 353 | (30) |  |
|  | Unknown | 39 | (4) |  |
| Histology | |  |  |  |
|  | Adenocarcinoma | 1149 | (99) |  |
|  | Adenosquamous | 16 | (1) |  |
| Gene status | |  |  |  |
|  | EGFR-mutated | 576 | (49) |  |
|  | ALK- rearranged | 67 | (5.7) |  |
|  | Pan-WT | 495 | (43) |  |
|  | ROS1- rearranged | 27 | (2.3) |  |
