# Supplementary material for: A Prediction Model for ROS1-Rearranged Lung Adenocarcinomas based on Histologic Features
Source: PLoS One. 2016 Sep 20;11(9):e0161861. doi: 10.1371/journal.pone.0161861 (PMC5029801; doi:10.1371/journal.pone.0161861)
Supplement: S2 Table — (DOC) [file pone.0161861.s006.doc]

Table S2. Sensitivity and Specificity of ROS1 IHC for ROS1 rearrangement by FISH Calculated on the Basis of 2+ Protein Expression Cutoff

|  | IHC2-3 + | IHC 0-1+ | Total |  |
| --- | --- | --- | --- | --- |
| FISH positive | 27 | 0 | 27 | Sensitivity=100% |
| FISH negative | 18 | 304 | 322 | Speciﬁcity=94.4% |
| Total | 45 | 304 | 349 |  |
