# Supplementary material for: A Prediction Model for ROS1-Rearranged Lung Adenocarcinomas based on Histologic Features
Source: PLoS One. 2016 Sep 20;11(9):e0161861. doi: 10.1371/journal.pone.0161861 (PMC5029801; doi:10.1371/journal.pone.0161861)
Supplement: S3 Table — (DOC) [file pone.0161861.s007.doc]

| Table S3.Pathologic Features of ROS1-rearranged NSCLCs | | | | | | | | | | |
| --- | --- | --- | --- | --- | --- | --- | --- | --- | --- | --- |
| Case | | Sex | Age | Smoking status | Dominant histologic pattern | Secondary histologic pattern | Mucinous and /or signet ring feature | Solid | Psammomatous calcification | Differentiation |
| **Resected group** | | | |  |  |  |  |  |  |  |
| 1 | Male | | 60 | Smoker | Papillary | Solid,Micropapillary | Little | Little | Yes | Moderate-low |
| 2 | Male | | 50 | Smoker | Acinar(cribriform) | Solid,Papillary | Yes | Yes | Yes | Moderate-low |
| 3 | Female | | 78 | never | Solid | Acinar(cribriform),Lepdic | No | Yes | No | Low |
| 4 | Female | | 70 | never | Acinar(cribriform) | Solid | Yes | Yes | Yes | Moderate-low |
| 5 | Female | | 59 | never | Acinar(cribriform) | Solid,signet-ring cell | Yes | Yes | No | Moderate-low |
| 6 | Male | | 54 | Smoker | Solid | Acinar(cribriform),Micropapillary,Lepdic | No | Yes | Yes | Low |
| 7 | Female | | 65 | never | Solid,signet-ring cell | Acinar(cribriform) | Yes | Yes | No | Low |
| 8 | Female | | 73 | never | Papillary | Solid (signet-ring cell), Acinar(cribriform),Lepdic | Yes | Yes | No | Moderate-low |
| 9 | Female | | 58 | never | Acinar(cribriform) | Solid,Micropapillary | Yes | Yes | Yes | Moderate-low |
| 10 | Female | | 57 | never | Lepdic | Solid | No | No | No | well-Moderate |
| 11 | Male | | 31 | never | Acinar(cribriform) | No | Yes | No | No | Moderate |
| 12 | Female | | 53 | never | Acinar(cribriform) | Solid | Yes | Yes | Yes | Moderate-low |
| 13 | Female | | 58 | never | Acinar(cribriform) | Solid | No | Yes | No | Moderate-low |
| 14 | Female | | 55 | never | Acinar(cribriform) | Solid | Yes | Yes | No | Moderate-low |
| **Advanced group** | | | |  |  |  |  |  |  |  |
| 1 | Female | | 45 | never | Solid | Micropapillary | No | Yes | No | Low |
| 2 | Female | | 38 | never | Solid | No | No | Yes | No | Low |
| 3 | Female | | 65 | never | Papillary | Micropapillary | No | No | No | Moderate |
| 4 | Male | | 27 | never | Unknown | Unknown | Unknown | Unknown | Unknown | Low |
| 5 | Male | | 56 | Smoker | Solid,signet-ring cell | No | Yes | Yes | No | Low |
| 6 | Female | | 46 | never | Solid,signet-ring cell | No | Yes | Yes | No | Low |
| 7 | Female | | 49 | never | signet-ring cell | No | Yes | Unknown | Unknown | Low |
| 8 | Female | | 39 | never | Acinar(cribriform) | No | Yes | Unknown | No | Moderate |
| 9 | Male | | 60 | Smoker | Solid | No | No | Yes | No | Low |
| 10 | Female | | 41 | never | Solid | No | No | Yes | No | Low |
| 11 | Male | | 31 | never | Solid | No | No | Yes | No | Low |
| 12 | Female | | 52 | never | Solid | No | Yes | Yes | No | Low |
| 13 | Female | | 58 | never | Papillary | No | No | No | No | Moderate |
